# Outcomes of near confluent laser versus combined less dense laser and bevacizumab treatment of prethreshold ROP Type 1 Zone 2: a randomized controlled trial

**DOI:** 10.1186/s12886-022-02689-0

**Published:** 2022-11-28

**Authors:** Ehsan Namvar, Alireza Bolkheir, Zahra Emadi, Mohammadkarim Johari, Mohammad Hossein Nowroozzadeh

**Affiliations:** Poostchi Ophthalmology Research Center, Department of Ophthalmology, School of Medicine, Shiraz University of Medical Sciences, Zand Street, Shiraz, 7134997446 Fars Iran

**Keywords:** Retinopathy of prematurity, Retinal laser therapy, Bevacizumab, Combination therapy

## Abstract

**Background:**

To evaluate the results of near confluent laser therapy versus combined less dense laser and intra vitreal bevacizumab in treatment of infants with type 1 retinopathy of prematurity (ROP) in zone II.

**Methods:**

This is a prospective double-blinded randomized clinical trial study. Infants with Type 1 ROP in Zone 2 were randomized into case and control groups. Conventional laser therapy was executed for control group and combination of IVB and laser treatment was employed for the case group.

**Results:**

Eighty-six eyes from 43 infants were analyzed in this trial. The first group included 42 eyes from 21 infants receiving a combination of laser ablation and IVB. The second group contained 44 eyes from 22 infants who received only conventional laser therapy. The combined IVB and laser ablation group demonstrated the neovascularization regression (20 out of 21 infants) one week after the procedure. In the conventional laser therapy group, this regression was found in (12 out of 22 infants) within one week after laser therapy (*P* = 0.001). Plus disease regression was observed in 20 (20/21) of combined treatment group and 7 infants (7/22) of conventional laser treatment group after one week.

**Conclusion:**

Combined less dense laser and bevacizumab treatment resulted in more rapid regression in comparison with the conventional laser treatment.

**Trial registration:**

IRCT20201120049450N1, 27/12/2021.

## Background

Retinopathy of prematurity (ROP) is an important factor for visual impairments in premature infants secondary to premature development of retinal vasculature [1]. In developed countries, it is recommended to screen cases with gestational age < 32 weeks and birth weight < 1500 g [2–5]. Although spontaneous regression would happen in the early stages, some cases may progress to harsh manifestations, leading to retinal haemorrhage and tractional retinal detachment necessitating surgical interventions. Noticeably, ROP management is currently the main concern of the Maternal and Child Health Care Organization [6–11]. A contemporary standard therapy for this problem is ablating the peripheral avascular area through creating laser photocoagulation scars where there are no skip areas [11–13]. The description of Type 1 high-risk pre-threshold ROP includes any ROP plus disease in Zone I, stage 3 ROP in Zone I, and stage 2 or 3 ROP with plus disease in Zone II [14, 15].

On the other hand, overtreatment may also cause adverse effects such as exudative retinal detachments, vitreous hemorrhages, and choroidal bleeding [16]. Both posterior and anterior segment complications may arise due to either indirect or direct impacts of laser ablation therapies because of sustaining laser burns [17–20]. In rare cases, there have been reports regarding anterior segment hemorrhage [21]. Further, posterior synechiae resulting from mild to moderate inflammation can intricate the therapy. Moreover, anterior segment ischemia, as a severe complication, causes developing hypotony, corneal opacification, cataract, pupillary membranes, or phthisis [19–23].

In addition to a better anatomical result, intravitreal bevacizumab (IVB) monotherapy is accompanied by fewer refractive errors in Zones I and II ROP [24, 25]. Nevertheless, this monotherapy leads to some disadvantages such as late reactivation, a persistent avascular peripheral retina, and disproved follow-up protocol [26, 27].

Regarding previous studies, the combination of IVB and Zone I sparing laser ablation as the primary therapy for treating Type 1 ROP in Zone I showed significantly better anatomical and functional results compared to laser treatments alone [25, 28, 29].

Conquering the mentioned disadvantages of both main treatments of ROP separately would compel researchers to evaluate combined treatments in various studies, and they have approved good anatomical outcomes in Zone I ROP without neither reactivation nor retreatment [30–36].

Concerning the poor anatomical results for Zone I ROP in comparison to Zone II ROP and a lack of studies about combined IVB and less dense laser in ROP type 1 Zone II, this clinical trial was performed on Zone II ROP. In addition, considering reactivation in the form of increased peripheral retinal vascularization months after IVB, the study forced on using laser therapy, along with IVB in ROP type 1 [37–40].

The present study evaluated the results of laser therapy individually and in combination with therapies in infants with Type 1 ROP in Zone II, as diagnosed by the Early Treatment for Retinopathy of Prematurity (ETROP) [16–18].

## Methods

A prospective double-blinded randomized clinical trial study was conducted on premature infants at Poostchi Eye Institute, Shiraz University of Medical Sciences.

Potential bevacizumab, injection, and laser-related side effects were explained to parents before obtaining informed consent, and all parents agreed to participate in the research and informed consent were obtained from their parents. Patients were randomized by stratified randomization method into case and control groups, and randomization was performed by an assistant who had no role in treating the patients. The trial ID in the Iranian Registry of Clinical Trials was IRCT20201120049450N1, 27/12/2021.

Neonates with ROP criteria and born from November 2020 until September 2021 were evaluated and incorporated into our work, along with those who had Type 1 ROP in Zone II. To detect a regression time of extraretinal fibrovascular proliferation and plus disease which is in agreement with the study of Banach et al. [41] with a two-sided 5% significance level and a power of 80%, 86 eyes from 43 infants with Type 1 ROP in Zone 2 were analyzed in this trial. Patients were randomized into case and control groups, and randomization was performed by an assistant who had no role in treating the patients.

A combination of IVB and laser treatment was employed for the first group as case group and conventional laser therapy was executed for the second group as the control group. Threshold ROP, ROP stage 4–5, vitreous hemorrhage, neovascularization of iris, aggressive posterior ROP, and elevated ridge were excluded from the investigation.

Treatment interventions, clinical course, complications, and anatomical results were evaluated after the treatment.

After preparation with 10% iodine/povidone solution and insertion of a lid speculum, bevacizumab (0.625 mg; 0.025 ml of StivantR, CinnaGen Co., Iran) was injected 1.5 mm posterior to the limbus by the application of the 30-gauge needle.

Near-confluent laser photocoagulation (laser diode photocoagulation DC-3300 NIDEX CO.LTD Japan) was conducted for 360° on the avascular retina using an 810-nm with mean power 350 ± 55mW, laser time 0.15 s and interval 0.3 s in the control group (*n* = 23). In the case group (*n* = 21), IVB injection and less dense laser (same protocol with laser spots placed 1 burn width apart) photocoagulation were exerted on the avascular retina for 360°. Treatments were performed in the operating room of the Ophthalmology Department at Khalili Hospital under general anesthesia while neonatal intensive care unit equipment was reserved.

Different ocular complications were evaluated, including cataract, hyphema, retinal detachment, retinal and vitreous hemorrhage, retinal fold, macular dragging, retrolental membrane, and vitreous organization (white fibrous opacification of vitreous over the avascular/vascular junction). Furthermore, the other evaluated complications were corneal edema, strabismus, nystagmus, phthisis bulbi, angle-closure glaucoma, and endophthalmitis. Follow-ups were weekly conducted until the regression of extraretinal fibrovascular proliferation and plus disease. Follow-ups were then continued every 2–4 weeks up to the age of three months (corrected) and then bimonthly until 6 months of age. The description of progression was developing threshold ROP, stage 4A, 4B, or 5 ROP. Next, laser treatment was repeated with the identification of skip areas, progression, or reactivation. Progression, reactivation, retreatment, pupillary reaction, and IOP, as well as the regression time of extraretinal fibrovascular proliferation and plus disease, and the number of laser spots in each eye were compared between the two groups.

Statistical analysis was conducted by SPSS to compare case and control groups using Mann–Whitney and Chi-square tests and the t-test, and *P*-values < 0.05 were statistically significant.

## Results

Totally, 86 eyes from 43 infants with Type 1 ROP in Zone 2 were analyzed in this trial. The first group included 42 eyes from 21 infants receiving a combination of laser ablation and IVB. The second group contained 44 eyes from 22 infants who received only conventional laser therapy. The average gestational age of the participants was 29.66 ± 2.08 and 30.31 ± 2.62 weeks in the first and second groups, respectively. The mean birth weight of the participants was 1277.61 ± 332.11 gr and 1457.95 ± 411.13 gr in the first and second groups, respectively. No statistically significant differences were noted in the mean birth weight (*P* = 0.185), gender (*P* = 0.172), and mean gestational age (*P* = 0.370) between the study groups. The baseline features of the research groups are provided in Table 1.

**Table 1:** Baseline characteristics of type 1 ROP infants treated with IVB plus laser therapy vs. laser therapy alone

Characteristic	Group		Mean difference (95% CI)
	IVB plus laser therapy	Laser therapy alone	
Patients, n	21	22	
Eyes, n	42	44	
GA, (mean ± SD), w	29.7 ± 2.1	30.3 ± 2.6	-0.7 (-2.1 to 0.8)
BW, (mean ± SD), g	1278 ± 332	1458 ± 411	-180 (-411 to 50)
Gender (Male/female)	10/11	15/7	

*95% CI* 95% confidence interval, *BW* birth weight, *GA* gestational age, *IVB* intravitreal bevacizumab, *SD* standard deviation

The mean number of laser spots was 1488.47 ± 198.54 and 1753.04 ± 152.30 in Groups I and II. Statistically significant differences were found between the study groups in terms of the number of laser spots (*P* = 0.000).

Based on the results, the mean baseline IOP in the first and second groups was 10.61 ± 0.97 and 11.09 ± 1.34 mmHg, respectively (*P* = 0.283). Additionally, the mean IOP, one day after the procedure, was 10.38 ± 1.35 and 10.68 ± 1.12 mmHg in the first and second groups, respectively (*P* = 0.262).

The combined IVB and laser ablation group demonstrated the neovascularization regression (20 out of 21 infants) one week after the procedure. In the conventional laser therapy group, this regression was found in 12 out of 22 infants within one week after laser therapy (*P* = 0.001).

In Groups I and II, the plus disease regression was observed in 20 (20/21) and 7 infants (7/22) one week after the procedure, respectively (*P* = 0.000).

The group receiving combined IVB and laser ablation showed the neovascularization and plus disease regression in all infants three weeks after the procedure. In control group, it was noticed in 20 infants (20/22) three weeks after laser therapy (*P* = 0.096). Finally, all infants represented ROP regression in this group 24 weeks after laser therapy.

Table 2 presents the regression of neovascularization and plus disease within 1, 2, 3, 4, and 24 weeks after the procedure of the two groups.

**Table 2:** Comparison of the regression of neovascularization and plus disease between studied groups during week 1 to week 24 after the treatment

Time	Outcome	Group		*P* value
		IVB plus laser therapy, n (%)	Laser therapy alone, n (%)	
Week 1	Neovascularization	20 (95%)	12 (55%)	0.001
	Plus Disease	20 (95%)	7 (32%)	< 0.001
Week 2	Neovascularization	20 (95%)	16 (73%)	0.036
	Plus Disease	20 (95%)	12 (55%)	0.001
Week 3	Neovascularization	21 (100%)	20 (91%)	0.49
	Plus Disease	21 (100%)	20 (91%)	0.49
Week 4	Neovascularization	21 (100%)	21 (95%)	0.243
	Plus Disease	21 (100%)	21 (95%)	0.243
Week 24	Neovascularization	21 (100%)	22 (100%)	
	Plus Disease	21 (100%)	22 (100%)	

*IVB* intravitreal bevacizumab

Based on the findings, no recurrence of ROP occurred in the two groups until 6-month follow-up. Pupillary membrane, anterior segment ischemia, vitreous hemorrhage, lens opacity, retinal detachment, or endophthalmitis were found in no infant at the last follow-up.

## Discussion

In the present work, the outcomes were compared in randomized participants with Type 1 ROP in Zone II treated with two different approaches, namely, a combination of less dense laser treatment and IVB, and conventional laser photocoagulation.

However, previous research reported the ROP reactivation even as late as 69 weeks' postmenstrual age following IVB [37]. The worries regarding late recurrences can be minimized by combined laser therapy.

In the study by Banach et al., the mean difference of the number of laser spots between the dense and less dense laser groups was 185. This study represented that the progression rate in the near confluent laser therapy group was 3.6% compared to 29% in the less dense laser therapy group [41]. In our study, although the mean difference of the number of laser spots between the dense and less dense laser plus IVB groups was 275, the rate of regression was similar and even the combination therapy group resulted in more rapid regression in comparison with the dense laser group.

In addition, IVB with less dense laser therapy can protect more visual fields compared to the conventional laser treatment.

Although laser photocoagulation permanently leads to neovascularization regression, it increases vascular endothelial growth factor (VEGF) temporarily [42, 43]; therefore, it develops with a 2–3 weeks delay. Meanwhile, ROP may progress during this time despite full laser treatment.

Although the effects of intravitreal anti-VEGFs do not last a long period, they immediately cause the regression of neovascularization [43, 44].

Accordingly, a combination of IVB and less dense laser has immediate and long-lasting effects, which perfectly explains the good results of this study. Further, previous studies demonstrated IVB as an adjuvant treatment [44–46].

Laser causes retinal atrophy and photoreceptor damage [28, 36], thus a less dense laser strategy seems to be less destructive and has better outcomes with less complications, and may allow the avascular retina to be vascularized in future instead of complete ablation and absolute visual field defects.

The BEAT-ROP study represented that the interval between bevacizumab injection and the reactivation of ROP was 19.2 ± 8.6 weeks [47]. Therefore, our follow-up period was enough to evaluate the recurrences.

In their study, Seo et al. found no neurodevelopmental delay in the IVB and laser combination therapy group in the long term [48].

In a meta-analysis by Popovi et al., there were no significant differences in the regression rate between IVB and dense laser; however, a higher retreatment rate was observed in the IVB group. Similarly, no differences were detected in safety outcomes between IVB and dense laser. IVB was associated with less surgical intervention and better refractive outcomes [49]. Accordingly, IVB and less dense laser combination therapy is safe and has less complications compared with dense laser treatment. These results are in agreement with our outcomes.

To the best of our knowledge, the current study is the first comparative randomized clinical trial conducted on IVB and less dense laser combination therapy and conventional laser therapy in patients with Type 1 ROP in Zone II.

The comparison of refractive outcomes, long-term side effects, and neurodevelopemental outcomes between the two groups will be evaluated in an extension study in future.

One of the limitations of our study was that it failed to control factors such as indomethacin or surfactants related to ROP between groups. [50–53].

## Conclusion

Combined less dense laser and bevacizumab treatment is safe and can result in more rapid regression in comparison with the dense laser. The rate of regression was similar between case and control groups.

## Data Availability

The datasets generated and/or analyzed during the current study are not publicly available due to the confidentiality of patients' information but are available from the corresponding author on reasonable request.

## Abbreviations

ROP: Retinopathy of prematurity
IVB: Intravitreal bevacizumab
ETROP: Early Treatment for Retinopathy of Prematurity
VEGF: Vascular endothelial growth factor
